# Fabrication of TiB_2_–Al1050 Composites with Improved Microstructural and Mechanical Properties by a Liquid Pressing Infiltration Process

**DOI:** 10.3390/ma13071588

**Published:** 2020-03-30

**Authors:** Seongmin Ko, Hyeonjae Park, Yeong-Hwan Lee, Sangmin Shin, Ilguk Jo, Junghwan Kim, Sang-Bok Lee, Yangdo Kim, Sang-Kwan Lee, Seungchan Cho

**Affiliations:** 1 Composites Research Division, Korea Institute of Materials Science (KIMS), Changwon 51508, Korea; ksm0901@kims.re.kr (S.K.); leeyh@kims.re.kr (Y.-H.L.); p996305s@kims.re.kr (S.S.); jhwankim@kims.re.kr (J.K.); leesb@kims.re.kr (S.-B.L.); 2 School of Materials Science and Engineering, Pusan National University, Busan 46241, Korea; 3 Advanced Materials Engineering, Dong-Eui University, Busan 47340, Korea; guswodld@naver.com (H.P.); ijo@deu.ac.kr (I.J.)

**Keywords:** Al matrix composite, Titanium diboride, infiltration, wettability

## Abstract

This study was conducted on titanium diboride (TiB_2_) reinforced Al metal matrix composites (MMCs) with improved properties using a TiB_2_ and aluminum (Al) 1050 alloy. Al composites reinforced with fine TiB_2_ at volume ratios of more than 60% were successfully fabricated via the liquid pressing infiltration (LPI) process, which can be used to apply gas pressure at a high temperature. The microstructure of the TiB_2_–Al composite fabricated at 1000 °C with pressurization of 10 bar for 1 h showed that molten Al effectively infiltrated into the high volume-fraction TiB_2_ preform due to the improved wettability and external gas pressurization. In addition, the interface of TiB_2_ and Al not only had no cracks or pores but also had no brittle intermetallic compounds. In conclusion, TiB_2_–Al composite, which has a sound microstructure without defects, has improved mechanical properties, such as hardness and strength, due to effective load transfer from the Al matrix to the fine TiB_2_ reinforcement.

## 1. Introduction

The demand for lightweight materials with specific performances for such applications as automobiles, aerospace technology, and armor, has gradually increased. Many studies on aluminum matrix composites (AMCs) have been conducted, because AMCs fabricated by adding ceramic reinforcements, such as silicon carbide (SiC) [1], alumina (Al_2_O_3_) [2], boron carbide (B_4_C) [3], titanium carbide (TiC) [4], titanium diboride (TiB_2_) [5], and hybrid [6] to an aluminum (Al) matrix, have excellent mechanical properties while maintaining low densities. Among the many ceramic reinforcements used in the fabrication of AMCs, TiB_2_ is a very attractive reinforcement due to its superior Young’s modulus [7], excellent hardness [8], and chemical stability with Al [9].

Most of the processes used in recent decades in the manufacturing of metal matrix composites (MMCs) are ex-situ methods in which reinforcement materials are added from the outside to the base material. Compared to in-situ methods, ex-situ processes make it easier to control the volume ratio of reinforcements and are advantageous in terms of cost and productivity. However, ex-situ methods have a limitation in that, because the reinforcement is physically bonded to the base, the interface between the reinforcement and the base is unstable [7,10,11]. Therefore, many researchers have implemented additional processes to compensate for interface defects, the main disadvantage of ex-situ methods. Urena et al. used a heat-treatment method that oxidized the surface of SiC reinforcements to improve the wettability with molten Al [12]. Rajan et al. coated nickel (Ni) or copper (Cu) on carbon/graphite, SiC, and Al_2_O_3_ to improve the wettability with the Al matrix [13]. Zhou et al. applied a Ni coating to TiC to improve the wettability between TiC and Al–Cu prior to manufacturing TiC_p_–Al–Cu composites [14]. Although these surface pre-treatment processes improve the soundness of the interface between a reinforcement and a matrix, they may not only change the composition of the base metal but also require extra time and cost. Kou et al. reported that the surface tension of Al linearly decreased as temperature increased [15]. Indeed, as the temperature increases, the wettability of TiB_2_ and Al increases. In particular, when temperature is over 1000 °C, the wettability increases to allow for spontaneous infiltration [16,17]. Therefore, if the process temperature is increased, wettability between reinforcements and the matrix easily increases, even without additional surface pre-treatment process. However, composites fabricated by only spontaneous infiltration process have partially non-infiltrated pores between reinforcements and the matrix. There have been some studies to solve this problem, including the removal of non-infiltrated pores through an additional gas pressurization process [18,19]. 

For this paper, we fabricated uniformly dispersed, high-volume fraction TiB_2_–Al composites through the liquid pressing infiltration (LPI) process, which is capable of fabricating MMCs at high temperature (up to 1800 °C) using applied gas pressure. The effects of the fine TiB_2_ reinforcements on the mechanical properties of Al composite were analyzed. If uniformly dispersed, fine TiB_2_–Al composites, which have an ideal microstructure with high hardness and high strength, can be fabricated by a cost-effective melting process, they will be highly useful in many industries, such as automobile, aerospace, and defense ones.

## 2. Experimental

### 2.1. Materials and Methods

To optimize the experimental conditions before fabricating the TiB_2_–Al composites, the sessile drop method was used to measure the contact angle between Al and TiB_2_ by dropping molten Al on a TiB_2_ plate (20 × 20 × 2 mm^3^) at 700–1000 °C under vacuum. Al1050 alloy (Portland Aluminum, Portland, Australia) was selected as the matrix material to minimize the effects of additive elements. The chemical composition of Al1050 is expressed in Table 1.

TiB_2_ powders (Kojundo Chemical Laboratory Co., Ltd., Saitama, Japan) with an average particle size of 2–3 μm were used as the reinforcement. TiB_2_ powders were compressed under a uniaxial pressure of 80 MPa, and were sintered at 1000 °C for 60 min under argon atmosphere to fabricate a porous TiB_2_ preform with a diameter of 50 mm and a height of 20 mm. The porosity of the fabricated TiB_2_ preform was calculated by measuring the volume and weight of the preform. TiB_2_–Al composites were fabricated by the LPI process, which was developed to infiltrate molten metal into a ceramic preform via hydrostatic pressure, which leads to a uniform dispersion of reinforcement inside the metal matrix [20]. The TiB_2_ preform and Al1050 were inserted into a magnesium oxide (MgO) crucible and heated to 1000 °C at a heating rate of 10 °C/min in a vacuum atmosphere (2.8 × 10^−1^ torr). After holding for 1 h at the target temperature, 10 bar of argon gas was injected to assist infiltration of the molten Al alloy into the porous TiB_2_ preform to fabricate the TiB_2_–Al composites.

### 2.2. Characterization

Microstructures of the TiB_2_–Al composites fabricated by LPI process ere analyzed by scanning electron microscope (SEM, JSM-6610LV, JEOL, Tokyo, Japan), transmission election microscope (TEM, JEM-ARM200F, JEOL, Tokyo, Japan), and electron probe micro analyzer (EPMA, JXA-8530F, JEOL, Tokyo, Japan). X-ray diffraction (XRD, D/Max-2500, Rigaku, Tokyo, Japan) using Cu Kα radiation at 30 kV and 250 mA was used to analyze the phase of the TiB_2_–Al composite. In addition, to calculate the relative density, the density of the composite was measured using the Archimedes method and compared with the theoretical density of the composite. Coefficient of thermal expansion (CTE) was measured from room temperature to 400 °C using a dilatometer (DIL 402C, NETZSCH, Selb, Germany). Room temperature tensile and compressive tests of TiB_2_–Al composites were carried out using a universal testing machine (5882 model, INSTRON, Norwood, MA, USA) at a strain rate of 5 × 10^−4^. In addition, a Vickers hardness tester (FM-700, Future-tech, Kanagawa, Japan) was employed to measure the hardness of the TiB_2_–Al1050 composites and Al1050 alloy at 300 KgF for 10 s. To confirm the reliability of the experiment, five tension samples, five compression samples, and three Vickers hardness samples were tested.

## 3. Results and Discussion

### 3.1. Contact Angle Between TiB_2_ and Al

**Figure 1:**
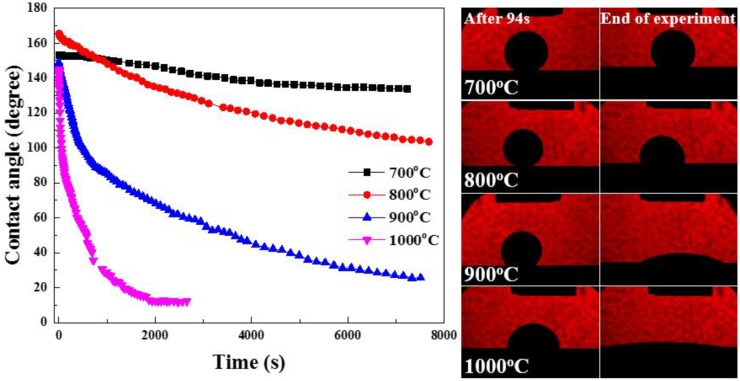
Contact angle between TiB_2_ substrate and molten Al.

To fabricate sound MMCs, it is very important to control the processing conditions—temperature, environment, etc. This experiment focused on the temperature, which increases the wettability between TiB_2_ and Al matrix in a vacuum. Contact angle measurement of TiB_2_ and Al was conducted up to 1000 °C in a vacuum to find the optimum processing conditions. Figure 1 shows the measured contact angles and provides images of the TiB_2_ plate and pure Al at 700, 800, 900, and 1000 °C.

The time is defined as the moment at which a droplet completely melts into a sphere; the spreading time is calculated from this point onward. The contact angles of Al and TiB_2_ at 700 and 800 °C were over 100°, even 6000 s after molten Al fell onto the TiB_2_ substrate, indicating the difficulty of spontaneous infiltration of molten Al into the TiB_2_ preform. On the other hand, the contact angles at 900 and 1000 °C dropped below 40°. In particular, the contact angle fell to 90° after 94 s of holding of the droplet on the TiB_2_ substrate at 1000 °C, indicating greatly increased wettability between TiB_2_ and Al. These results reveal that, at 1000 °C, molten Al can easily infiltrate into a porous TiB_2_ preform in a relatively short time. In this research, therefore, TiB_2_–Al1050 composites were fabricated at 1000 °C using the LPI process.

### 3.2. Morphology of TiB_2_ Preform

**Figure 2:**
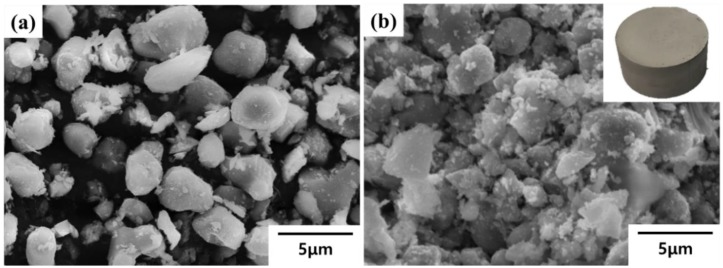
SEM images of (**a**) TiB_2_ power and (**b**) TiB_2_ preform.

The uniform dispersion of reinforcement in the composite is considered to help effective infiltration of the molten metal alloy and improve the mechanical properties of MMCs. In this experiment, to fabricate uniformly dispersed homogeneous TiB_2_–Al composites, a TiB_2_ reinforcement was prepared in the form of a porous structure. As-received TiB_2_ powder has irregular shapes, as shown in Figure 2a. 

In addition, it was confirmed that the observed TiB_2_ particle size was in the range of 2–3 μm, although relatively large particles of about 5 μm and small debris particles several nm in size also existed. In this experiment, TiB_2_ powders were sintered at 1000 °C, which is much lower than the sintering temperature of TiB_2_ ceramic [21], to form weakly bonded TiB_2_ preforms. The inset of Figure 2b shows the pre-sintered TiB_2_ preform at 1000 °C; it has a diameter of 50 mm and a height of 20 mm. It is important for successful infiltration of molten Al during the LPI process that the ceramic preform maintains the shape of the porous body by slight bonding between ceramic particles, without grain growth. An SEM image of the sintered TiB_2_ preform shows a porous microstructure with 2–3 μm sized TiB_2_ particles, indicating no grain growth after sintering at 1000 °C (Figure 2b). TiB_2_ particles were weakly bonded to each other while maintaining their origin shapes, resulting in the generation of about 35 vol.% porosity. Consequently, at elevated temperature, molten Al is effectively infiltrated into the uniformly generated open pores in the TiB_2_ preform.

### 3.3. Microstructure of TiB_2_–Al1050 Composites

**Figure 3:**
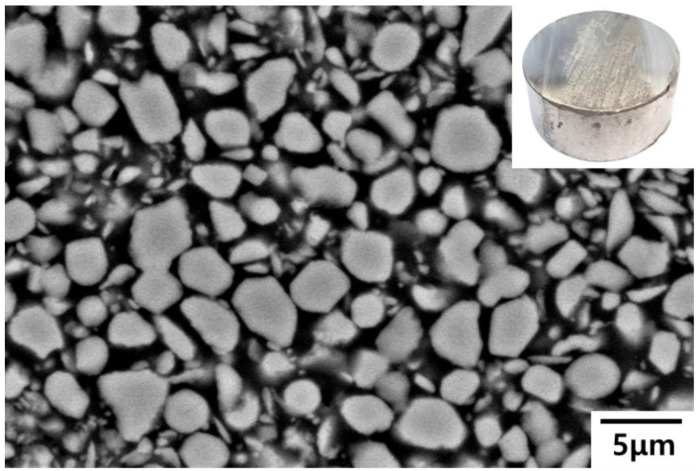
Microstructure of TiB_2_–Al1050 composites fabricated by LPI process.

Figure 3 provides SEM and OM images of the TiB_2_–Al composite fabricated by LPI process. After the infiltration of molten Al into the TiB_2_ preform, the color changed from gray to silver (inset of Figure 3). 

Due to the effective infiltration of molten Al into the porous TiB_2_ preform during the LPI process with the aid of applied gas pressure, TiB_2_ reinforcements did not aggregate; rather, they individually dispersed in the Al1050 matrix. The volume fraction of the TiB_2_ reinforcement, analyzed using the Image J program on several SEM images taken from the center and side area of the sample, was about 65%, which proves that the Al substrate was ideally infiltrated into the porous TiB_2_ preform, which had a porosity of about 35%. In addition, the particle size of the TiB_2_ reinforcement in the TiB_2_-Al composite fabricated by LPI process is similar to the particle size of raw TiB_2_. Therefore, it was confirmed that no grain growth of the TiB_2_ reinforcement occurred at 1000 °C during the LPI process.

**Figure 4:**
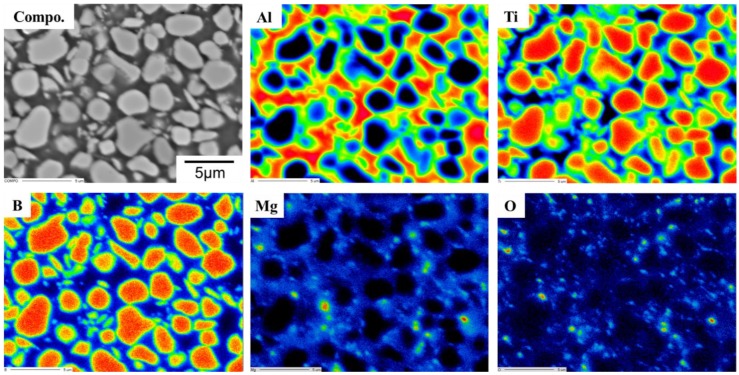
EPMA mapping images of TiB_2_–Al1050 composites.

Figure 4 shows experimental results of EPMA for composition analysis of TiB_2_–Al composites fabricated by LPI process. 

The element mapping image indicates that Ti and B atoms were detected only at the TiB_2_ particle sites. The Al and TiB_2_ regions can be clearly distinguished. TiB_2_ reinforcements in the composites were observed to be homogeneously dispersed, without aggregation, despite the high volume ratio. From the Al mapping image, it was confirmed that molten Al infiltrated not only the micro-sized places between TiB_2_ reinforcements having average particle sizes of 2–3 μm, but also the narrow places between TiB_2_ reinforcements of sub-micron size. If wettability between the ceramic reinforcement and metal matrix is poor, infiltration of molten metal will be more difficult in high volume fraction MMCs than in low volume fraction MMCs because of the increase of interfacial area and the high surface energy. This result proves that the superior wettability between TiB_2_ and Al was actually obtained by fabrication of high volume fraction composite at the process temperature of 1000 °C. On the other hand, a few Mg-related oxides were observed in the microstructure of the TiB_2_–Al composites. This was probably generated by the reactions of minor elements in Al1050 with additional Mg and O from the MgO crucible. However, the number of phases was extremely low, such that the effect of these phases on the mechanical properties of TiB_2_–Al composites was negligible.

**Figure 5:**
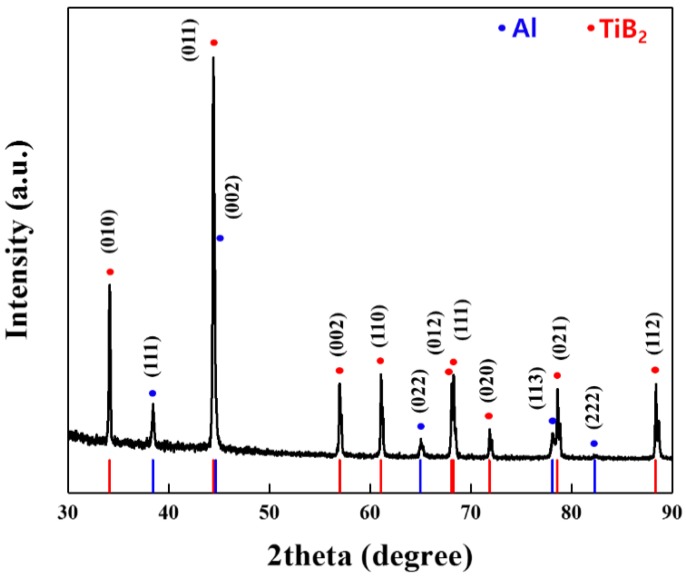
XRD pattern of TiB_2_–Al1050 composites.

Figure 5 shows the results of XRD analysis of TiB_2_–Al composites, allowing identification of the reaction phase formed during the fabrication process.

Generally, TiB_2_ is more thermodynamically stable than intermetallic compounds such as AlB_2_ and Al_3_Ti, as well as other ceramics such as B_4_C and TiC with Al [22,23]. Therefore, no interfacial reaction between TiB_2_ and the Al matrix occurred during the LPI process. XRD results for TiB_2_–Al composites fabricated by LPI process at 1000 °C showed only Al and TiB_2_ peaks. Although some Mg oxide was found in the EPMA mapping images, the XRD results do not indicate any Mg oxide peaks, indicating the generation of an extremely small amount of Mg oxide in the Al matrix. 

**Figure 6:**
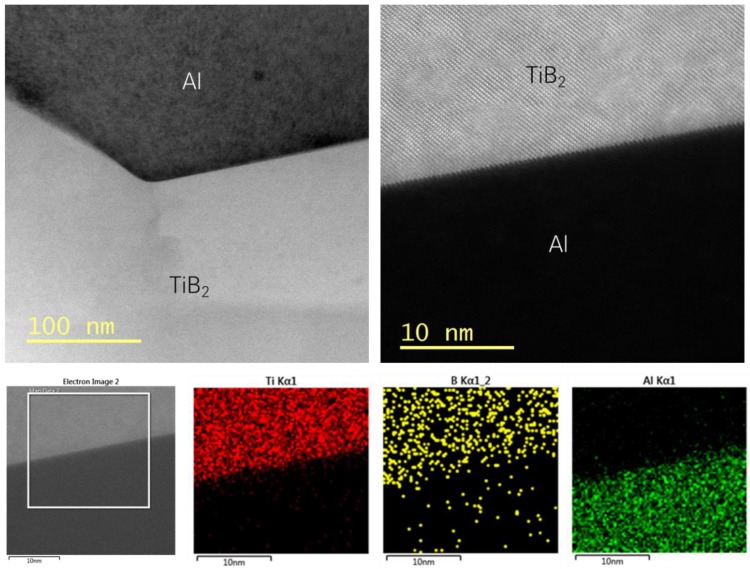
TEM images and EDS mapping images of TiB_2_–Al1050 composites.

The microstructures of the TiB_2_–Al composites fabricated by LPI process were analyzed using TEM, as indicated in Figure 6. 

The microstructure of the TiB_2_–Al composite was found to be an extremely clean TiB_2_/Al1050 interface without any vacancies or defects. It can be assumed that the TiB_2_/Al interface is likely to have a semi-coherent nature. These results reveal that, due to the improved wettability between TiB_2_ and Al at a high processing temperature and the Ar gas pressurization applied, molten Al effectively infiltrated the TiB_2_ preform. In addition, the clean TiB_2_ particles show that they formed a sound interface without damaging the reinforcement during the fabrication process, despite the difference in CTE between TiB_2_ (6–8 ppmK^−1^) and the Al matrix (26.28 ppmK^−1^). Figure 6 also provides energy-dispersive X-ray spectroscopy (EDS) element mapping images of the TiB_2_/Al1050 interface, allowing an analysis of the formation of chemical compounds at the interface between TiB_2_ and Al. As mentioned earlier, because it is chemically very stable, TiB_2_ is expected not to form chemical compounds during the composite fabrication process [22,23]. Actually, the results of EDS analysis of the TiB_2_/Al1050 interface indicate no chemical reaction between TiB_2_ and the Al1050 matrix at 1000 °C, implying that the TiB_2_–Al composites fabricated in this experiment do not have extremely brittle compounds such as Al_3_Ti or AlB_2_, which are formed through chemical reactions [24,25,26]. Consequently, the TiB_2_–Al composites fabricated by LPI process, having sound microstructures without brittle compounds, indicate the possibility of achieving mechanical properties better than those of other composites fabricated by other ex-situ processes.

### 3.4. Mechanical Properties of TiB_2_–Al1050 Composites

**Figure 7:**
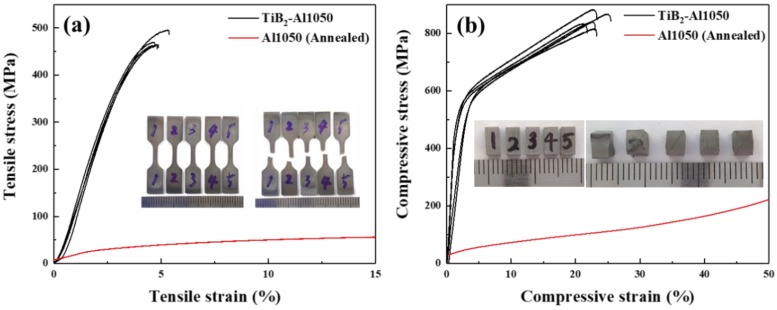
(**a**) Tensile and (**b**) compressive stress–strain curves of TiB_2_–Al1050 composite and Al1050, tested at room temperature.

Figure 7 shows the tensile and compressive stress–strain curves of TiB_2_–Al composites tested at room temperature. 

The insets of the figure are images of TiB_2_–Al composite specimens taken before and after the test. In the graph, the black line indicates the TiB_2_–Al1050 composite and the red line indicates the Al1050 alloy. Due to the influence of the strong and brittle TiB_2_ reinforcement, the strength of the TiB_2_–Al composite was significantly improved, while elongation decreased compared with that of the Al1050 alloy. As can be seen from the slope of the tensile stress–strain curve, the elastic modulus of the composite seems to be significantly improved compared to Al1050 due to the high elastic modulus (530 GPa) of TiB_2_ [7]. In general, it is difficult to measure the tensile strengths of ceramics due to their brittle nature, which was confirmed by fracturing in the elastic deformation region during the tensile test at room temperature. High volume fraction ceramic reinforced MMCs also exhibit the same fracture behavior as normal ceramics. Therefore, stress–strain curves of MMCs usually show fractures in the elastic deformation region [27,28,29]. However, TiB_2_–Al1050 composites fabricated by the LPI process showed not only slight plastic deformation in the tensile test but also obvious plastic deformation behavior in the compression test. This phenomenon is very interesting and requires precise analysis. If the fine reinforcement is uniformly dispersed in the metal matrix while maintaining a good interface with the matrix, the tensile strength of the composite will increase without severe elongation deterioration. The results reveal that fine TiB_2_ dispersed Al MMCs having sound interface were successfully fabricated by LPI process by increasing infiltration temperature and with the aid of applied pressure.

Table 2 summarizes mechanical and physical properties, such as density, hardness, strength, and CTE values of the TiB_2_–Al1050 composites and the Al1050 alloy, respectively.

Due to the relatively high density of TiB_2_ (4.52 g/cm^3^), the density of the TiB_2_–Al1050 composite increased 1.4 times compared to that of the Al1050 alloy. However, the ultimate tensile strength (UTS) of the TiB_2_–Al1050 composite dramatically increased to 7.0 times compared with Al1050. The tensile strength of Al1050 at room temperature was 67.1 MPa, as shown in Table 2. However, the average tensile strength of the TiB_2_–Al composites was 471.5 MPa, which was significantly high.

The compressive yield strength (CYS) of the TiB_2_–Al1050 composite also increased about 8.4 times compared with that of the Al1050 matrix. In addition, Vickers hardness showed an 8.4-times increase compared with that of the Al1050 matrix. The CTE value of the TiB_2_–Al1050 composite, measured from room temperature to 100 °C, was 12.97 ppmK^−1^, which is about 49% lower than that of Al1050. The characteristics of the composites might be anticipated by considering approximate values obtained using the rule of mixtures (ROM), which can be used to determine the characteristics and compositions of mixed materials [30]. The rule of mixtures follows this formula:

(1) $$\sigma_{c}=\sum \left(f_{i}\cdot \sigma_{i}\right)=f_{1}\cdot \sigma_{1}+f_{2}\cdot \sigma_{2}+\cdots +f_{n}\cdot \sigma_{n}$$

Most of the measured properties of the TiB_2_–Al1050 composites fabricated by LPI process were within the ranges of properties calculated based on ROM. However, the tensile strength of the TiB_2_–Al1050 composite is extraordinarily higher than those of monolithic bulk TiB_2_ and Al. This phenomenon is very interesting, as mentioned in the results of the stress–strain curve showing the plastic deformation behavior of the composites. The tensile strength of bulk ceramics is usually low due to premature failure; there are no standard values because the strength of a bulk ceramic depends on the grain size and distribution of defects. This result is believed to be due to the synergistic effect of the fine microstructure and the sound interface between TiB_2_ and the Al1050 matrix.

**Figure 8:**
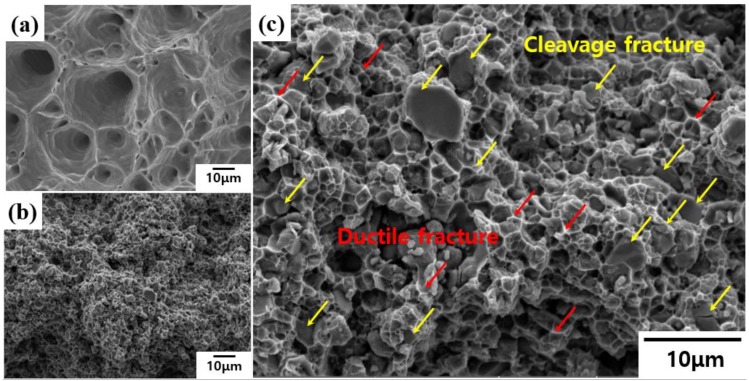
Fractography of (**a**) Al1050 and (**b**,**c**) TiB_2_–Al1050 composite specimens after tensile tests.

Fracturing through tensile load commonly starts at defects such as voids. In the case of ceramic-reinforced metal matrix composites, when tension is applied in the vertical direction to composites in which soft Al, having low strength, is present, the material stretches, and fractures proceed as defects are formed at interface between reinforcements and the matrix. If this interface is not sound, the reinforcements–matrix interface plays a major role in the formation of defects such as voids [31]. Therefore, composites having poor interfaces may exhibit poor mechanical properties due to interfacial delamination. Figure 8a,b shows the fracture surfaces after tensile testing of Al10150 and TiB_2_–Al1050 composites, respectively, under the same magnification.

The fractography of Al10150 shows that, when load was applied to the specimen in the vertical direction, the specimen broke while forming coarse dimples of several tens of micrometers. In the case of the TiB_2_–Al1050 composite, the microstructure became very fine compared with that of the Al matrix; this was due to the presence of fine TiB_2_ reinforcement and the grain refined Al matrix. Figure 8c shows the microstructure observed at high magnification to allow for the analysis of the fracture surface of the TiB_2_–Al1050 composite; significantly fine dimples and lots of broken TiB_2_ particles can be seen. This fracture behavior appeared when cracks formed at the soft matrix were effectively transferred to the reinforcements. Through this phenomenon, the TiB_2_–Al1050 composite fabricated by LPI process is considered to exhibit fracture behavior in which brittle fracturing of TiB_2_ and ductile fracturing of Al are mixed by effective load transfer, without interfacial delamination between TiB_2_ and Al [28]. Consequently, highly improved tensile strength and moderate ductility of TiB_2_–Al1050 composites were achieved by using the LPI process at 1000 °C and the aid of applied gas pressure to reinforce a high-volume fraction of fine TiB_2_ in the Al matrix.

## 4. Conclusions

In this study, fabrication of TiB_2_–Al composites with improved mechanical properties and microstructures was attempted to compensate for the unstable interfaces of composites fabricated by ex-situ methods. Using the advantage of improved wettability between TiB_2_ and Al with increasing temperature, TiB_2_–Al composites having uniformly dispersed fine TiB_2_ reinforcements at about 65% volume ratios were successfully fabricated using the LPI process, which applies gas pressure at a high temperature. At the high temperature of 1000 °C, molten Al1050 alloy was well infiltrated into the very narrow sub-micron regions between TiB_2_ particles; no interfacial defects were produced due to the good wettability between TiB_2_ and the Al matrix and the aid of the applied gas pressure. The density of the composite was 3.84 g/cm^3^, which is 1.4 times higher than that of the matrix. The tensile strength, CYS, and Vickers hardness of the TiB_2_–Al composite were 471.5 MPa, 500.4 MPa, and 194.4 Hv, respectively, which are 7.0, 8.4, and 8.4 times higher than those values of Al1050. In addition, CTE of the composite is about 49% lower than that of Al1050 in the range of room temperature to 100 °C. Because of the absence of brittle intermetallic compounds and interfacial defects in the TiB_2_–Al composite, the load applied to the composite was effectively transferred from the soft Al matrix to the high strength TiB_2_ reinforcement, resulting in mixed fracture behavior, including ductile and brittle fracturing. For this reason, tensile and compressive tests show that the strength and elongation of TiB_2_–Al composites are significantly improved compared to those values of conventional high-volume fraction MMCs fabricated by ex-situ methods. Therefore, with their excellent mechanical and physical properties, TiB_2_–Al composites fabricated by LPI process are considered applicable in various fields, such as those pertaining to automobiles, aerospace, and armor.

## Figures and Tables

**Table 1:** Composition of Al1050 alloy.

Al1050	Al	Fe	Cu	Mg	Mn	Si	Ti	V	Zn
Composition	99.8% ≥	0.1% ≤	0.001% ≤	0.001% ≤	0.02% ≤	0.05% ≤	0.04% ≤	0.02% ≤	0.002% ≤

**Table 2:** Measured mechanical and physical properties of TiB_2_–Al1050 composite and Al1050.

Specimens	Density (g/cm^3^)	UTS (MPa)	CYS (MPa)	Hardness (Hv)	CTE (ppmK^−1^)
TiB_2_–Al1050	3.84	471.5	500.4	194.4	12.97
Al1050	2.71	67.1	59.4	23.2	26.28
